# When Disability Recognition Fails: Accommodation Refusals and Socioeconomic Challenges Among Canadians with Multiple Chemical Sensitivity

**DOI:** 10.3390/ijerph23081076

**Published:** 2026-08-19

**Authors:** Nene A. Diallo, John Molot, Riina Bray, Adrianna Trifunovski, Rohini Peris

**Affiliations:** 1 Association pour la Santé Environnementale du Québec—Environmental Health Association of Québec (ASEQ-EHAQ), Saint-Sauveur, QC J0R 1R1, Canada; ndiallo@aseq-ehaq.ca (N.A.D.); adrianna@aseq-ehaq.ca (A.T.); 2 Faculty of Medicine, University of Ottawa, Ottawa, ON K1G 5Z3, Canada; jmolot@rogers.com; 3 Environmental Health Clinic, Women’s College Hospital, Toronto, ON M5S 1B2, Canada; riina.bray@rogers.com; 4 Department of Family and Community Medicine, University of Toronto, Toronto, ON M5G 1V7, Canada

**Keywords:** multiple chemical sensitivity, disability accommodation, socioeconomic challenges, housing instability, employment barriers, institutional recognition, environmental disability, Canada

## Abstract

**Highlights:**

**Public health relevance—How does this work relate to a public health issue?**

- Multiple chemical sensitivity (MCS) can limit access to safe employment, housing, healthcare, and other essential determinants of public health, contributing to broader health inequities.
- This study examines how barriers to accommodation affect the social, economic, and health outcomes of Canadians living with MCS.

**Public health significance—Why is this work of significance to public health?**

- High rates of accommodation refusal, unemployment, unsafe housing, and homelessness among people with MCS highlight the substantial public health burden associated with inadequate disability recognition and support.
- The findings demonstrate how institutional barriers can contribute to social exclusion and worsen health outcomes among individuals with MCS and related disabilities.

**Public health implications—What are the key implications or messages for practitioners, policy makers and/or researchers in public health?**

- Employers, housing providers and public spaces should implement accommodation measures that prioritize reduced levels of chemical exposures so that people with MCS can safely access these environments.
- Public health policies and disability frameworks should recognize the accessibility needs of those with MCS to promote health equity, housing stability, and workforce participation.

**Abstract:**

Background: Multiple chemical sensitivity (MCS), a recognized disability, is a chronic, multisystem condition in which exposure to common chemical substances provokes adverse health effects and functional impairment. Although population-level data and emerging clinical research support the pathophysiological basis of MCS, individuals living with the condition frequently encounter barriers to recognition and accommodation across employment, healthcare, and housing systems. This study examines patterns of accommodation requests, refusals, and associated socioeconomic impacts among adults living with MCS in Canada, with particular attention to how institutional recognition shapes access to supports. To our knowledge, this is the first national Canadian study to examine accommodation request outcomes across both employment and housing among adults with MCS, combining quantitative and qualitative data. A national cross-sectional survey of 119 Canadian adults living with MCS was conducted between January and February 2021. Quantitative data were analyzed descriptively to assess accommodation requests, outcomes, employment status, income, and housing stability. Qualitative responses were analyzed thematically to contextualize participants’ experiences of disclosure, and accommodation outcomes. Participants were predominantly female (87%) with post-secondary education (90%). Among participants who reported being unemployed (29%), 94% attributed it primarily to MCS-related limitations. Accommodation denial was common: 85% of respondents reported requesting accommodations, and 78% of those experienced refusals. On housing conditions, 52% reported living in unsafe housing (defined as housing with the presence of mould or symptom-triggering exposures), while 17% reported being homeless (e.g., moving frequently, personal vehicle, tent). Findings should be interpreted in light of the cross-sectional design, self-reported data, and community-based recruitment, which may limit generalizability. The findings suggest that inconsistent institutional recognition of MCS can function as a barrier to accommodation, contributing to health, economic, and social inequities. Addressing these barriers requires clarified guidance on the duty to accommodate multiple chemical sensitivity, proactive scent-free and lowest-emission policies in workplaces and housing, and improved healthcare provider education to support accommodation documentation.

## 1. Introduction

Multiple chemical sensitivity (MCS) is a recognized disability, which is increasing in prevalence [1,2,3,4,5]. It is a chronic, multisystem condition in which exposure to low levels of common chemical substances leads to recurrent adverse health effects. Triggers frequently include fragranced consumer products, cleaning agents, pesticides, building and renovation materials, and vehicle exhaust [5,6,7]. A widely cited conceptual model describes MCS as involving an initial sensitizing exposure, followed by symptom elicitation upon subsequent exposures at levels tolerated by the general population [8,9,10]. Clinical manifestations are heterogeneous and may involve neurological, respiratory, gastrointestinal, cardiovascular, endocrine, and musculoskeletal systems [11,12]. For many individuals, symptoms fluctuate in relation to environmental exposures, resulting in substantial functional impairment and constraints on daily activities, employment, and social participation [13,14].

Over the past two decades, a growing body of international research has strengthened evidence for the biological and pathophysiological basis of MCS, advancing understanding of its mechanisms, symptom patterns, and impacts on health and quality of life [9,10]. Despite this expanding literature, recognition of MCS within healthcare systems remains inconsistent. Individuals with MCS frequently encounter skepticism, diagnostic uncertainty, and fragmented care pathways, which can delay access to appropriate management and exacerbate illness trajectories [13,15,16]. These barriers are not limited to clinical interactions but extend into broader institutional contexts where medical recognition functions as a prerequisite for access to support and accommodations.

Population-level data indicate that MCS represents a significant and growing public health concern in Canada. Analyses of Statistics Canada’s Canadian Community Health Survey (CCHS), synthesized and reported by the Association pour la Santé Environnementale du Québec—Environmental Health Association of Québec (ASEQ-EHAQ), indicate that more than 1.3 million Canadians were living with diagnosed MCS by 2020 [5,14,17]. Preliminary Statistics Canada data from January–July 2025 further suggest that approximately 3.1 million Canadian adults (9.4%) reported MCS, while approximately 900,100 adults (2.7%) report receiving a medical diagnosis [18]. In addition, individuals with MCS experience disproportionate health complexity: 77.8% report at least one additional chronic condition, whereas 45.0% of the general population report comorbidity [19]. Prevalence of specific comorbidities is markedly elevated, including asthma (25.7% vs. 8.3%) and non-food allergies (61.1% vs. 26.6%) [20]. These patterns underscore the cumulative health burden associated with MCS and the corresponding need for responsive care and accommodation systems.

Despite its prevalence and complexity, MCS remains under-recognized and under-resourced. Individuals frequently report consulting multiple healthcare providers before finding one with sufficient knowledge of the condition, a process that is financially and logistically burdensome and may exclude those with fewer resources [21]. Compounding this challenge, healthcare and institutional environments often involve routine exposure to chemical triggers, such as fragranced products, disinfectants, and cleaning agents, creating additional barriers to accessing care [21]. In Ontario, the Task Force on Environmental Health estimated that more than 740,000 residents live with MCS and two other comorbidities, describing these conditions as “profoundly life altering”. The Task Force concluded that MCS remains poorly recognized and inadequately managed within the health-care system, citing shortages of knowledgeable providers, limited clinical tools, and insufficient services and support [22]. By 2020, diagnosed MCS cases exceeded 400,000 in Ontario alone [5,17].

In Canada, under the Canadian Human Rights Act (and provincial and territorial human rights legislation), employers, landlords, and service providers have a duty to accommodate persons with disabilities to the point of undue hardship [1]. Under this framework, the Canadian Human Rights Commission (CHRC) explicitly recognizes environmental sensitivities (including MCS), as a disability. At the provincial level, the Ontario Human Rights Commission has affirmed comparable protections [23]. In practice, however, accommodation is approved on a case-by-case basis and most often requires medical documentation validating both the disability and the requested measures. For individuals with MCS, this requirement conflicts with inconsistent clinical recognition and low healthcare provider knowledge on the condition [21,22], further complicating efforts to obtain the required documentation. Furthermore, the nature of the accommodation further distinguishes MCS from other conditions. Indeed, whereas adjustable workstations or entryways may be more apparent, MCS-related accommodations require the cooperation of co-workers, neighbours, or entire organizations through sustained behavior. Perceptions of cost and complexity of implementation may thus appear, even where the legal duty to accommodate is clear. This accommodation landscape provides an essential context of interpreting the request and refusal patterns highlighted in this study.

Barriers associated with MCS extend beyond healthcare into employment, housing, and other domains of social participation. In systems where access to accommodation, such as fragrance-free workplaces or lowest-emission housing, depends on medical validation, lack of recognition can function as a structural barrier to inclusion [14,23]. As a result, individuals with MCS are frequently denied workplace accommodations [24], compelled to live in unsafe or unstable housing environments [25,26], and experience elevated risks of unemployment, poverty, and social isolation [6,15,27]. These outcomes occur despite high levels of education and work experience among many individuals with MCS [28], suggesting that environmental and institutional barriers, rather than individual capacity alone, contribute to these patterns.

Previous analyses based on the present dataset have examined environmental exposure pathways associated with MCS onset and symptom triggering, as well as the impacts of COVID-19 public health measures on healthcare access for individuals with MCS [12,16]. However, no national study has yet quantified accommodation request outcomes across both employment and housing among Canadian adults with MCS, or paired such outcomes with qualitative accounts of the accommodation process itself. Building on this foundation, the current study addresses this gap by examining the denial of disability accommodations in employment and housing contexts. Using quantitative and qualitative data from a national cross-sectional survey of Canadian adults living with MCS, this paper examines patterns of accommodation requests and refusals, perceived barriers to disclosure, and the socioeconomic consequences of denied access. By centering lived experience alongside population-level indicators, the study illuminates how institutional recognition and legitimacy shape accommodation outcomes and contribute to structural inequities affecting people with MCS. The analysis is informed by the social model of disability, which locates disadvantage in environments and institutions that fail to remove barriers to participation rather than in impairment alone [29], and by the sociological literature on contested illnesses, which examines how conditions marked by medical uncertainty must be legitimized before entitlements attach [30]. Within this health equity perspective, the study considers both structural factors and individual experiences in interpreting accommodation outcomes.

## 2. Materials and Methods

### 2.1. Study Design

This study employed a cross-sectional survey design to examine accommodation experiences and socioeconomic impacts among Canadian adults living with MCS. Data collection occurred between 19 January and 12 February 2021, during the COVID-19 pandemic. The survey included both current and retrospective questions, allowing participants to report experiences prior to and following the implementation of pandemic-related public health measures.

The timing of data collection is relevant to interpretation. Pandemic-related public health measures altered the accommodation landscape in both directions: expanded remote work reduced some workplace exposure and accommodation needs, while intensified cleaning and disinfection protocols increased chemical exposures in housing, services, and remaining in-person settings. For this reason, experiences were captured separately for the periods before and after 11 March 2020, and results are presented for both periods throughout.

The present analysis forms part of a broader research program examining barriers faced by individuals with MCS across social determinants of health. This paper focuses specifically on accommodation requests and outcomes in employment and housing settings, as well as perceived retaliation, disclosure-related concerns, and downstream socioeconomic effects.

### 2.2. Ethics Approval

This study received ethical approval from the Women’s College Hospital Research Ethics Board (Protocol #2020-0157-E). All study procedures complied with the principles of the Declaration of Helsinki. Participants provided informed consent electronically prior to participation. Survey responses were anonymized and de-identified to protect confidentiality. No financial incentives were provided.

### 2.3. Participants and Eligibility

Participants were eligible to take part if they:

- Were 18 years of age or older;
- Resided in Canada;
- Had experienced symptoms consistent with MCS for at least one year prior to 11 March 2020;
- Were able to complete the survey in English or French.

Eligibility was confirmed through a brief pre-screening questionnaire. Only individuals who met all criteria were directed to the full survey.

### 2.4. Data Collection and Recruitment

Participants were recruited using a national, multi-channel strategy that included organizational websites, social media platforms, targeted online advertising, and direct outreach through ASEQ-EHAQ mailing lists. Recruitment materials invited individuals living with MCS to share their experiences related to access, accommodation, and quality of life.

The survey was administered using the Qualtrics XM platform (version 2021; Provo, UT, USA). Participation was voluntary and anonymous. To reduce access barriers, respondents who were unable to complete the survey online were offered the option of telephone-based participation with trained interviewers.

A total of 373 individuals accessed the survey. Of these, 119 met inclusion criteria and completed the full questionnaire, forming the analytic sample for this study.

This recruitment approach constitutes a community-based convenience sample. Because participation required individuals to independently access and complete an online survey, the study may under-represent individuals with the most severe symptoms or functional limitations who may have been unable to participate despite being eligible. Participants were recruited primarily through the websites of the Environmental Health Clinic, Toronto, and ASEQ-EHAQ, including ASEQ-EHAQ’s email and social media channels. This recruitment strategy may also have resulted in greater representation from Québec, where ASEQ-EHAQ is based. Accordingly, the findings should not be interpreted as representative of all Canadians living with MCS, and the implications for generalisability are discussed in the Limitations.

### 2.5. Survey Instrument

The survey comprised 84 items, of which 16, relevant to this analysis, were assessed. These included experiences with accommodation processes in employment and housing, such as whether accommodation was requested, the outcomes of those requests, and reasons for not seeking accommodation. Other questions captured changes in employment status, household income, housing conditions, and perceived socioeconomic impacts of accommodation outcomes (File S1).

Survey items were developed collaboratively with researchers, clinicians, and individuals with lived experience of MCS, and were pilot-tested with members of the MCS community to ensure relevance and content validity.

For more information regarding survey development, content validity, and reliability testing, refer to a related peer-reviewed paper [14].

### 2.6. Data Analysis

Quantitative data were analyzed descriptively using frequencies and percentages to characterize accommodation requests, refusal rates, employment status, income, and housing conditions. Where appropriate, subgroup comparisons were conducted to examine relationships between accommodation outcomes and socioeconomic indicators.

Qualitative responses were analyzed using thematic analysis. Open-ended responses were coded inductively to identify recurring patterns related to disclosure, accommodation denial, emotional impacts, and adaptive strategies. Coding was conducted by a trained qualitative researcher on the study team, with codes iteratively refined into candidate themes that were reviewed against the full dataset and discussed with the research team (NAD, RP, JM, AT), and individuals with lived experience. Credibility was supported through team review of themes, and the use of verbatim participant quotations. Qualitative findings were used to contextualize quantitative results and provide insight into the lived experience of navigating accommodation systems.

## 3. Results

### 3.1. Summary of Key Results

Across domains, the findings reveal persistent and interrelated barriers to safe participation in employment, housing, and healthcare. Despite high levels of education, participants reported elevated unemployment, low income, and housing insecurity, with more than half residing in environments that exacerbated MCS symptoms. Accommodation requests were frequent and often repeated, yet denials were common both before and during the COVID-19 pandemic. Fear of stigmatization, retaliation, and prior negative experiences further deterred accommodation seeking, particularly following the onset of pandemic-related public health measures. Housing instability emerged as a critical vulnerability, with increased chemical exposures linked to infection-control practices contributing to displacement and reduced access to safe living environments. Collectively, these findings describe frequent accommodation denial occurring alongside socioeconomic instability and housing precarity. These findings suggest that institutional practices (including reliance on medical validation and exposure-generating environments) may contribute to barriers to obtaining essential accommodations and to the socioeconomic and health challenges reported by participants living with MCS.

### 3.2. Participant Characteristics

#### 3.2.1. Sociodemographic Profiles

The analytic sample comprised 119 adults who self-identified as living with MCS and met all eligibility criteria, including being 18 years of age or older, residing in Canada, and having experienced MCS-related symptoms for at least one year prior to 11 March 2020.

Most respondents identified as women (87%, *n* = 103), while 13% (*n* = 16) identified as men. Participants ranged in age from 25 years to over 75 years, with 68% between 45 and 74 years of age. Respondents were primarily residents of Quebec (45%; *n* = 54) and Ontario (35%, *n* = 42). This distribution broadly reflects the concentration of Canada’s population in Ontario and Quebec, the country’s two most populous provinces, although Quebec residents were overrepresented relative to their share of the national population (45% versus approximately 23%), likely reflecting recruitment through a Québec-based organization (Table 1).

#### 3.2.2. Socioeconomic Characteristics

More than half of respondents (57%, *n* = 68) held a university degree, and 39% (*n* = 46) were employed. Among participants who were unemployed (29%, *n* = 34), 94% (*n* = 32) attributed their job loss directly to limitations associated with MCS. Other chronic conditions (such as fibromyalgia) were also reported as a reason for unemployment by 56% of respondents (*n* = 19) (Table 2). The most commonly reported annual household income category was $20,000 CAD or less (26%, *n* = 31).

### 3.3. Housing Characteristics and Residential Stability

Regarding housing conditions, 48% (*n* = 57) reported living in what they considered to be safe housing—defined as environments that minimize exposure to chemical triggers such as fragrances, mould, renovation materials, emissions from neighbouring units, or nearby traffic and industrial sources. Conversely, 52% (*n* = 62) indicated that their current housing did not meet these criteria.

Most participants were residing in conventional housing arrangements, including owned, rented, or subsidized housing at the time of the survey (83%, *n* = 99), whereas 15% (*n* = 18) described living situations involving frequent relocation, temporary stays with friends or family, personal vehicles, or tents. Regarding living arrangements, 42% (*n* = 50) reported living with 1 other person, while 39% (*n* = 46) reported living alone (Table 3).

### 3.4. Accommodation Requests and Outcomes

Among the 119 participants, the majority (85%, *n* = 101) reported having submitted at least one accommodation request in the year prior to 11 March 2020, for their disability. Among those, just over three-quarters of participants (76%, *n* = 77) reported submitting five or more requests, and at least 78% were denied at least once (Table 4).

### 3.5. Reported Reasons for Not Requesting Accommodations

Participants who reported not having requested accommodations were asked to identify factors contributing to this decision. Prior to 11 March 2020, 18 respondents provided reasons for not pursuing accommodation requests. The most commonly cited reasons were prior negative experiences when requesting accommodations and fear of being stigmatized (each 44%, *n* = 8). Remaining deterrents included concern about offending others, perceived lack of understanding or support related to MCS, and fear of retaliation (each 39%, *n* = 7). Fear of verbal abuse was also reported by one-third of participants (33%, *n* = 6).

Following 11 March 2020, 50 respondents reported reasons for not pursuing accommodation requests. The most frequently cited barriers during this period were prior negative experiences when requesting accommodations and concerns about losing respect if perceived as difficult (each 50%, *n* = 25), followed by perceived lack of understanding of MCS within support systems and fear of verbal abuse (each 46%, *n* = 23). Fear of losing professional support (44%, *n* = 22) and fear of being stigmatized (44%, *n* = 22) were also prominent (Table 5).

### 3.6. Qualitative Themes: Experiences of Accommodation Denial

The qualitative data were analyzed using a disability-access lens, focusing on how institutional norms, credibility assessments, and power asymmetries shape accommodation outcomes. Five interrelated themes were identified:

- Theme 1: Legitimacy as a Gatekeeper to Accommodation: Participants repeatedly described accommodation systems that require medical or institutional validation before access needs are acknowledged. In the absence of formal recognition of MCS, requests for environmental accommodations were routinely dismissed, delayed, or ignored.

*“Because my health condition is not recognized and I feel I don’t have any rights.”*

Through participants’ accounts, access appeared to depend more on whether a condition is perceived as legitimate within medical or bureaucratic hierarchies, rather than on functional need. Even when symptoms were severe, participants described being unable to translate lived experience into recognized entitlement. This dynamic contextualizes the quantitative finding that the majority of participants who requested accommodation reported at least one refusal (Table 4).

- Theme 2: Cycles of Exposure and Repeated Refusal: Rather than isolated denials, participants described repeated cycles in which exposure to triggering environments was followed by accommodation requests, which were then refused—often multiple times.

*“Even though I asked for accommodation, I was always terrified, always scared and feared for my safety. Sometimes people would increase the activity.”*

*“Too afraid to ask for help or accommodation. People sometimes increase what they are doing because I asked them to stop.”*

These accounts reveal how denial was not passive: In some cases, participants reported that requests for safer environments were followed by increased exposure, intensifying both health risks and emotional distress. These accounts help explain the repeated accommodation requests documented quantitatively: just over three-quarters of participants who sought accommodation before the pandemic had submitted five or more requests (Table 4).

- Theme 3: Anticipated Harm and Strategic Non-Disclosure: Many participants reported choosing not to request accommodation due to fear of retaliation, loss of housing or employment, or social hostility. This anticipatory harm shaped behaviour even in the absence of explicit refusal. Consistent with this pattern, the proportion of participants who did not request accommodations increased from 15% before March 2020 to 42% afterwards (Table 4), and fear-related deterrents were among the most frequently cited reasons for not seeking accommodation (Table 5).

*“The abuse taken for having asked for accommodation, stigma, privacy concerns, likelihood of refusal.”*

- Theme 4: Social Withdrawal as a Form of Self-Protection: Some participants described withdrawing from social, professional, or housing-related interactions as a protective strategy to reduce exposure and avoid repeated conflict. Rather than continuing to request accommodations, individuals limited engagement altogether, accepting isolation as a trade-off for safety. This adversity was described by participants as emotionally exhausting, limiting their capacity to advocate for accommodation.

While this strategy reduced immediate exposure, it often came at the cost of social participation, reinforcing isolation and exclusion.

*“I don’t interact with people much; when I do I wear a mask; so I don’t have to ask for accommodation. This has relieved me of a lot of anxiety!”*

- Theme 5: Emotional Exhaustion and Advocacy Fatigue: Repeated denials, combined with ongoing health challenges, resulted in profound emotional fatigue. Participants described reaching a point where continued self-advocacy was no longer sustainable.

*“…always worried about repercussions and if I would have the energy to ask for the 10th time…”*

The advocacy fatigue described here parallels the finding that nearly half of participants who requested accommodations before the pandemic reported five or more refusals (Table 4).

Table 6 summarizes the five themes identified in the thematic analysis, along with their defining characteristics.

## 4. Discussion

### 4.1. Summary of Findings

In this study, accommodation experiences and socioeconomic characteristics among individuals living with MCS were examined by conducting a national survey with qualitative accounts. Findings highlighted that accommodation requests were frequent (asked 5 times or more), and often refused (5 times or more), a pattern that was similar across both employment and housing contexts. Despite high education attainment among participants, most reported unemployment (attributed to MCS), low income and unsafe/unstable housing. Qualitative quotes further suggest that such barriers could be shaped by the processes through which accommodation needs are assessed and granted.

### 4.2. Theoretical Framework: Social Model of Disability and Contested Illness

The social model of disability locates disability not in the impairment itself, but in the interaction between individuals and societal, institutional, and environmental barriers that restrict full and effective participation in society [29,31]. This perspective is reflected in the United Nations Convention on the Rights of Persons with Disabilities (UN CRPD), which recognizes that disability results from the interaction between persons with impairments and attitudinal and environmental barriers. Within this framework, the duty to accommodate, embedded in Canadian human rights legislation, requires employers, healthcare providers, housing providers, educational institutions, and other duty holders to take reasonable steps to remove or reduce barriers and provide accommodations, rather than requiring individuals with disabilities to adapt to inaccessible environments, up to the point of undue hardship [1].

In practice, however, access to accommodation often depends on medical verification of the disability. Although the legal right to accommodation is based on the functional impact of a disability rather than scientific consensus regarding its etiology, accommodation processes typically require medical documentation confirming both the disability and the requested measures. For individuals with MCS, whose clinical status continues to be contested in some settings, this reliance on medical validation can create substantial barriers to accessing accommodations. As one participant explained:

*“Because my health condition is not recognized and I feel I don’t have any rights.”*

In this study, participants frequently described accommodation as contingent on their illness first being accepted as legitimate (Theme 1). Many expressed a sense that they had to establish legitimacy before entitlement to accommodation would follow. The absence of medical consensus, however, may force individuals living with MCS into a protracted social struggle for legitimacy, credibility, and institutional recognition, a pattern consistent with the concept of epistemic injustice, whereby a person’s account of their own condition is discounted because of the contested nature of the illness [32]. Consistent with the sociology of contested illness, this may also help explain the repetition documented in the survey: just over three-quarters of participants who requested accommodation before the pandemic submitted five or more requests, and nearly half were refused five or more times (Table 4). However, when the process used to verify legitimacy requires an answer to a medical question that the law itself does not require, individuals may experience exclusion because of how the accommodation process is designed, rather than because the evidence supporting their functional limitations is lacking. In Canada, although MCS is recognized as a disability under Canadian human rights law and by the Canadian Human Rights Commission, the condition remains poorly recognized in clinical practice [33,34].

### 4.3. Why Accommodation Requests Fail: The Mechanisms

The repeated unsuccessful accommodation requests can be explained by three reinforcing mechanisms. First, as discussed in the previous section, the duty to accommodate often depends on medical documentation. As clinical recognition of MCS remains inconsistent [21,33], many individuals living with MCS may be unable to obtain the medical validation that accommodation processes require. Second, exposure-related barriers differ fundamentally from more readily identifiable barriers, such as architectural barriers, whose removal often involves a one-time physical modification. Exposure-related barriers often arise from routine activities and occupant behaviour, are dispersed throughout environments rather than confined to a single location and are frequently invisible to others. Consequently, their removal requires sustained behavioral compliance by all parties (e.g., adherence to scent-free policies). In this context, employers and landlords may perceive additional implementation challenges, particularly with respect to policy monitoring, enforcement, and resistance to compliance [35]. Third, limited awareness of MCS among human resources personnel, landlords, healthcare providers, and other service providers (as reported by participants; Table 5) represents a significant barrier to accommodation. If a service provider has little understanding of MCS as a disability-related need, an accommodation request may be interpreted as a personal preference rather than a disability-related need, for which there is no perceived legal obligation to act. This may be further compounded by the contested status of MCS.

Other explanations also warrant consideration. First, requests involving broader environmental changes (such as measures affecting an entire workplace or building) may be refused more frequently than narrowly scoped requests. Second, participants reporting five or more accommodation requests were also those who persisted after initial refusals. Consequently, the high number of refusals documented may partly reflect continued requests following earlier denials, rather than the probability of any single request being refused. From the perspective of those responding to accommodation requests (although not directly examined in this study), MCS-related requests may present practical challenges, including uncertainty about effective accommodations due to limited knowledge of the condition and the absence of established professional guidance for MCS comparable to that available for mobility-related disabilities. Accordingly, some reported refusals may plausibly reflect these practical constraints rather than skepticism about the individual making the request.

Repeated refusals and anticipated harm, however, appear to suppress subsequent accommodation requests. The proportion of participants who did not request accommodation increased from 15% before March 2020 to 42% afterwards (Table 4), and fear-related reasons (including fear of retaliation and social consequences) were frequently cited. This reluctance to disclose accommodation needs may further reinforce the institutional invisibility of MCS, limiting opportunities for employers and service providers to gain experience in responding appropriately to future requests. More importantly, the increase in individuals not requesting for accommodation may reflect both deterrence and changing circumstances. During the pandemic, the expansion of remote work may have reduced the need to request workplace accommodations, rather than the willingness to do so, as working from home removed the need to request a scent-free workplace. Nevertheless, the predominance of fear-related reasons (Table 5) supports the interpretation that deterrence played a substantial role.

### 4.4. Systemic vs. Attitudinal Barriers

In this study, the identified barriers fall into two categories: systemic and attitudinal. Systemic barriers are embedded within institutions and their processes, whereas attitudinal barriers arise from interactions between people. In this context, inconsistent clinical recognition of MCS, the requirement for medical documentation, and the uncertainty regarding accommodation procedures for MCS suggest the presence of systemic barriers, reflected in the quantitative pattern of frequent accommodation refusals. These findings may indicate, for the most part, not the actions of individual decision-makers, but rather that standard accommodation procedures do not adequately account for conditions such as MCS.

Attitudinal barriers, on the other hand, were reflected in participants’ qualitative accounts, which described being disbelieved, resisted, or, in some cases, deliberately exposed to symptom-triggering substances after disclosing their condition. These experiences may help explain the fear and reluctance expressed by many participants, and may also contribute to the increase in non-requesting of accommodations from 15% to 42% (Table 4). More importantly, the distinction between these two types of barriers may be less clear in practice, as years of institutional non-recognition may eventually shape individual attitudes and behaviors. Accordingly, efforts to improve accommodation should address both types of barriers. For example, training staff to be more understanding is unlikely to be sufficient if the underlying institutional processes continue to provide no route to accommodation.

### 4.5. Comparison with National and International Literature

At a national level, the patterns identified in this study are consistent with, but more pronounced than, accommodation outcomes reported for the broader Canadian disability population. According to the 2017 Canadian Survey on Disability [36], two in five employees with disabilities who required workplace accommodations had only some (19%) or none (21%) of their needs met, and 40% of those with unmet needs who requested accommodation were refused. In 2022, more than one-third of employed Canadians with disabilities also reported an unmet accommodation need [37]. These comparisons suggest that individuals living with MCS may experience greater challenges obtaining accommodations than other disability groups.

A related consideration concerns the self-reported nature of participants’ attributions. The finding that 94% of unemployed participants attributed their job loss to MCS reflects participants’ understanding of the cause rather than an independently verified determination and is therefore subject to recall bias. Nevertheless, participants’ reports are consistent with evidence from the Canadian Community Health Survey demonstrating lower employment rates and lower incomes among Canadians living with MCS [28], strengthening, although not confirming, the inference that MCS contributes to adverse employment outcomes.

At the international level, these findings are also consistent with research from the United States examining workplace fragrance exposure and employment impacts. Studies have similarly reported job loss and reduced work capacity associated with fragrance exposure [7,38]. The consistency of these findings across different legal jurisdictions suggests that the barriers identified are not unique to a particular legal framework. However, because relatively few studies have examined accommodation outcomes specifically for individuals with MCS, opportunities for direct comparison remain limited. Further studies addressing this gap would strengthen future comparisons.

### 4.6. Limitations

This study presents some limitations worth highlighting. First, although the findings and interpretations describe a process involving accommodation requests, refusals, and subsequent deterrence, the cross-sectional design cannot observe this process unfolding over time. Second, the data were collected during the COVID-19 pandemic, and pandemic-related disruptions may have independently contributed to housing instability and employment outcomes (e.g., through atypical labour market conditions). Third, the findings rely on self-reported data from individuals with MCS and are therefore subject to recall bias. Unemployment reasons may additionally be subject to attribution bias, as discussed above. Furthermore, no data were collected from employers, landlords, clinicians, or human resources personnel. Accordingly, the interpretation of epistemic injustice is derived from one side of interactions involving at least two parties. Fourth, participants were recruited through the Women’s College Hospital and ASEQ-EHAQ websites, and ASEQ-EHAQ’s social media. This recruitment approach may have over-represented individuals connected to these networks, and residents of Québec. Conversely, individuals with the most severe illness may have been under-represented because the demands of completing an online survey may have limited their participation. Consequently, the findings may not be representative of Canadians living with MCS or the broader MCS population. Fifth, without a comparison group of individuals with other disabilities, this study cannot determine whether the challenges documented exceed those experienced by other disability groups. Although the findings can be compared with those of the Canadian Survey on Disability to provide useful context, differences in sampling and measurement limit direct comparison. Finally, future research comparing socio-economic outcomes with regional labour-market and housing data would help quantify the magnitude of the disparities suggested by the findings.

### 4.7. Implications

At an institutional level, implementation of proactive measures, such as scent-free policies, removes the need for individuals to first establish legitimacy of their condition before accommodations are provided. Systemic barriers are thereby reduced, and compliance with healthier indoor air practices becomes an organizational norm rather than an individual responsibility. This approach also advances health equity, as those most affected are often least able to absorb the economic consequences of exclusion, as reflected in the income and housing findings.

For employers, such measures would reduce occupant exposure while minimizing interpersonal conflicts associated with individually negotiated accommodation requests. For healthcare providers, further education on MCS, coupled with standardized documentation based on functional limitations rather than diagnostic certainty, could improve access to healthcare and enable physicians to support accommodation requests without requiring complete medical consensus. For landlords and housing providers, the use of lowest-emission products during cleaning and maintenance, advance notices of renovations and the consideration of MCS during unit allocation may significantly improve housing safety and stability. For policymakers, clearer guidance on implementing the duty to accommodate, together with the integration of MCS considerations into building standards and codes, could shift accommodation from case-by-case adjudication toward a more universal and proactive approach.

This study provides the first national Canadian examination of accommodation request outcomes across both employment and housing among adults living with MCS. By linking the frequency and outcomes of accommodation requests with the socioeconomic circumstances of this population, this cross-sectional survey identifies two key mechanisms—dependence on medical documentation and limited awareness among decision-makers—that may contribute to accommodation failures for individuals living with conditions that remain inconsistently recognized in clinical practice.

## 5. Conclusions

This study highlights the repeated challenges individuals with MCS face in securing accommodations across employment and housing. These challenges may lead to considerable adverse consequences, including economic instability, housing insecurity and social withdrawal.

Through an accommodation policy and public health lens, findings suggest that Canada’s accommodation framework, relying on individual medical validation, may under-serve any condition where clinical validation is unreliable (e.g., inconsistently recognized conditions). As such, proactive measures implemented at the institutional level (such as scent-free policies) would provide improved indoor air quality with benefits extending to all building occupants.

By documenting, for the first time in a Canadian sample, accommodation request outcomes among individuals with MCS and across both the employment and housing sectors, this study contributes to an under-researched area. Further research could include longitudinal designs examining accommodation outcomes and socioeconomic trajectories over time, as well as comparative studies involving other disability groups.

## Figures and Tables

**Table 1:** Sociodemographic Characteristics of Study Participants (N = 119).

Characteristic	Category	*n* (%)
Sex	Female	103 (87)
	Male	16 (13)
Age Group (years)	25–34	9 (8)
	35–44	23 (19)
	45–54	19 (16)
	55–64	43 (36)
	65–74	19 (16)
	75+	6 (5)
Province of Residence	Quebec	54 (45)
	Ontario	42 (35)
	Other provinces *	23 (19)

* “Other provinces” include Alberta, British Columbia, Nova Scotia, New Brunswick, Saskatchewan, PEI and Manitoba.

**Table 2:** Socioeconomic Characteristics of Participants.

Variable	Category	*n* (%)
Education (N = 119)	Secondary Only	9 (8)
	College/CEGEP	40 (34)
	University	68 (57)
	Prefer not to answer	2 (2)
Work Status (N = 119)	Employed	46 (39)
	Unemployed	34 (29)
	Retired or Student	39 (33)
Reason(s) for unemployment * (N = 34)	MCS condition	32 (94)
	Other ***	19 (56)
	COVID-19 health measures	8 (24)
Annual Income (N = 119)	≤$20,000	31 (26)
	$20,001–$40,000	17 (14)
	$40,001–$60,000	21 (18)
	$60,001–$80,000	14 (12)
	>$80,000	21 (18)
	No income	1 (1)
	Prefer not to answer	14 (12)

* Percentages may exceed 100% participants could select multiple options. *** The other category includes fibromyalgia and chemical exposure in the workplace.

**Table 3:** Housing Characteristics and Residential Stability.

Variable	Category	*n* (%)
Living in Safe Housing *	Yes	57 (48)
	No	62 (52)
Current Housing Situation	Housing **	99 (83)
	Homeless ***	18 (15)
	Other	2 (2)
Living Arrangement	Lives alone	46 (39)
	Living with 1 person	50 (42)
	Living with 2+ other people	20 (17)
	Prefer not to answer	3 (3)

Note: Percentages are based on respondents who answered each item. Due to non-response, totals may not equal the full sample (*n* = 119). * Safe housing refers to environments minimizing exposure to chemical triggers, including mould, renovation materials, exposures to triggers from neighbours, and nearby traffic or industrial emissions. ** Includes affordable housing, owner, renting. *** Includes moving frequently, personal vehicle, tent, or staying with friends/family.

**Table 4:** Accommodation Requests and Outcomes.

Variable	Category	Before 11 March 2020 *n* (%)	After 11 March 2020 *n* (%)
Requested accommodation (N = 119)	Yes	101 (85)	69 (58)
	No	18 (15)	50 (42)
Number of times accommodation was requested (N = 101|69)	1	4 (4)	9 (13)
	2	7 (7)	6 (9)
	3	5 (5)	14 (20)
	4	4 (4)	2 (3)
	5 times or more	77 (76)	28 (41)
	No answer	4 (4)	10 (14)
Reasonably accommodated (N = 101|69)	Yes	39 (39)	29 (42)
	No	59 (58)	30 (43)
	No answer	3 (3)	10 (15)
Number of times accommodation was refused (N = 101|69)	1	10 (10)	7 (10)
	2	7 (7)	5 (7)
	3	10 (10)	9 (13)
	4	4 (4)	1 (1)
	5 times or more	47 (47)	17 (25)
	No answer	23 (23)	30 (43)

Note: Percentages may not total 100% due to rounding.

**Table 5:** Reason (s) for not requesting accommodation.

Reason	Before 11 March 2020 *n* (%) N = 18	After 11 March 2020 *n* (%) N = 50
Bad experience when accommodation was previously requested	8 (44)	25 (50)
Fear of losing respect if perceived as difficult	7 (39)	25 (50)
Fear of verbal abuse	6 (33)	23 (46)
Lack of understanding of MCS from human resources	7 (39)	23 (46)
Fear of being stigmatized	8 (44)	22 (44)
Fear of losing professional support	4 (22)	22 (44)
Fear that others will feel their choices are being challenged	6 (33)	21 (42)
Fear of losing the little support I have	3 (17)	21 (42)
Fear of losing friends	5 (28)	20 (40)
Fear of emotional abuse	4 (22)	19 (38)
Fear of insulting others	7 (39)	19 (38)
Fear that the law is not on my side	4 (22)	18 (36)
Lack of understanding of MCS from police	5 (28)	17 (34)
Fear of retaliation (e.g., increased use of triggering products)	7 (39)	17 (34)
Fear of losing family	3 (17)	17 (34)
Lack of understanding of MCS from social workers	3 (17)	15 (30)
Fear of being abandoned	1 (6)	15 (30)
Other reasons	3 (17)	14 (28)
Fear of physical abuse	1 (6)	13 (26)
Other reasons related to COVID-19 health measures	1 (6)	11 (22)

Note: Percentages may exceed 100% participants could select multiple options.

**Table 6:** Thematic analysis of accommodation denial and its consequences.

Theme	Description
1. Legitimacy as Gatekeeper to Accommodation	Accommodation access contingent on medical or institutional recognition rather than functional need
2. Cycles of Exposure and Refusal	Repeated accommodation denials following ongoing chemical exposure
3. Anticipated Harm and Non-Disclosure	Fear of retaliation or social consequences deters accommodation requests
4. Social Withdrawal as Self-Protection	Avoidance of interaction to reduce exposure and conflict
5. Emotional Exhaustion and Advocacy Fatigue	Advocacy fatigue resulting from repeated denial and institutional resistance

## Data Availability

The data sets generated and analyzed during this study are available from the corresponding author upon reasonable request.

## Supplementary Materials

The following supporting information can be downloaded at: https://www.mdpi.com/article/10.3390/ijerph23081076/s1, File S1: Survey questions.
