# Supplementary material for: When Disability Recognition Fails: Accommodation Refusals and Socioeconomic Challenges Among Canadians with Multiple Chemical Sensitivity
Source: Int J Environ Res Public Health. 2026 Aug 19;23(8):1076. doi: 10.3390/ijerph23081076 (PMC13512570; doi:10.3390/ijerph23081076)
Supplement: Supplementary file 1 [file ijerph-23-01076-s001.zip › ijerph-4447843-supplementary.pdf]

Supplementary Materials—Survey Questions

Demographics

Q1. What is your age? *Required*

- Less than 18 *(end of survey)*
- 18–19
- 20–24
- 25–29
- 30–34
- 35–39
- 40–44
- 45–49
- 50–54
- 55–59
- 60–64
- 65–69
- 70–74
- 75–79
- 80 and over

Q2. State your province or territory of residence *Required*

- Alberta
- British Columbia
- Manitoba
- New Brunswick
- Newfoundland and Labrador
- Northwest Territories
- Nova Scotia
- Nunavut
- Ontario
- Prince Edward Island
- Quebec
- Saskatchewan
- Yukon
- Prefer not to answer

Q3. What is your sex? *Required*

- Female
- Male
- Other (please specify in the box below)
- Prefer not to answer

Q4. What is the highest level of education that you have completed? *Required*

- Less than secondary school graduation
- Secondary school graduation
- Post-secondary education (CÉGEP, College, trade school)
- Undergraduate degree (Bachelor's)
- Post-graduate certificate

- Master's degree
- Doctorate or Postdoctoral degree
- Prefer not to answer

Q5. Including yourself, how many people live in your household? *Required*

- 1
- 2
- 3
- 4
- 5
- 6 or more
- Prefer not to answer

Q6. What is your annual household income? *Required*

- No income
- Less than $5000
- $5000–$10,000
- $10,000 to $15,000
- $15,000 to $20,000
- $20,000 to $30,000
- $30,000 to $40,000
- $40,000 to $50,000
- $50,000 to $60,000
- $60,000 to $70,000
- $70,000 to $80,000
- Greater than $80,000
- Prefer not to answer

Q7. What is your work status?

- Employed
- Unemployed
- Self-employed
- Student
- Retired
- Prefer not to answer

Q8. The reason/s for your current unemployment is related to:

- COVID-19 health measures (disinfection, scented disinfection, physical distancing, wearing of gloves or masks, etc.)
- MCS condition
- Other reason (s)

Housing

Q9. Do you live in safe housing that is suitable for your MCS health condition?

(Safe housing is as follows and should be free of the following: mould, materials that trigger your symptoms (such as paint, new construction/renovation materials), exposures to triggers from neighbours or nearby emissions from traffic or industry, etc.*)*

- Yes

- No

Q10. What is your current housing situation? *Check off all that apply*

- Affordable/social housing
- Homeless
- Home or property owner
- Moving frequently
- Renting a property
- Shelter, drop-in centre, group home
- Tent
- Personal vehicle
- With friends or family
- Other

Disability Accommodation

Q11. Have you requested accommodation (s) for your disability? (e.g., scent-free products, least toxic disinfection etc.)

	**Yes**	**No**
Before 11 March 2020	☐	☐
After 11 March 2020	☐	☐

Q12. If you have not requested accommodation despite having exposures that you cannot avoid and which trigger symptoms, please select the reason (s) why:

Dropdown list:

- Bad experience when accommodation was asked for previously
- Fear of reprisal (verbal abuse)
- Fear of reprisal (emotional abuse)
- Fear of reprisal (physical abuse)
- Fear that the law is not on my side
- Lack of understanding of MCS from support systems (police)
- Lack of understanding of MCS from support systems (social workers)
- Lack of understanding of MCS from support systems (Human Resources)
- Fear of insulting people
- Fear that people will feel that their choices are being challenged
- Fear of retaliation (increased use of the product/activity that is triggering symptoms)
- Fear of being stigmatized
- Fear of losing friends
- Fear of losing family
- Fear of being abandoned
- Fear of losing the little support I have
- Fear of losing professional support (doctor, dentist, nurse, lawyer, etc.)

- Fear of losing respect if people think I am difficult (friends, family, professionals, etc.)
- Other reason (s) related to COVID-19 health measures
- Other reason (s)

Q13. Please specify the other reason (s) for not requesting accommodation before or after 11 March 2020:

(Open text field)

Accommodation Requests

Q14. How many times have you requested accommodation (s) for your disability?

Reminder: "before 11 March 2020" refers to 1 year before this date.

	**1 Time**	**2 Times**	**3 Times**	**4 Times**	**5 or More Times**
Before 11 March 2020	☐	☐	☐	☐	☐
Between 11 March 2020 to Present	☐	☐	☐	☐	☐

Q15. Were you reasonably accommodated following your request (s)?

	**Yes**	**No**
Before 11 March 2020	☐	☐
Between 11 March 2020 to Present	☐	☐

Q16. How many times have you been refused accommodation for your disability?

Reminder: "before 11 March 2020" refers to 1 year before this date.

	**1 Time**	**2 Times**	**3 Times**	**4 Times**	**5 or More Times**
Before 11 March 2020	☐	☐	☐	☐	☐
Between 11 March 2020 to Present	☐	☐	☐	☐	☐
